# Ethylene emitted by viral pathogen-infected pepper (*Capsicum annuum* L.) plants is a volatile chemical cue that attracts aphid vectors

**DOI:** 10.3389/fpls.2022.994314

**Published:** 2022-09-29

**Authors:** Sun-Jung Kwon, Soo-Jung Han, Myung-Hwi Kim, Seok-Yeong Jang, Ji-Soo Choi, Jang-Kyun Seo

**Affiliations:** ^1^ Institutes of Green Bio Science and Technology, Seoul National University, Pyeongchang, South Korea; ^2^ Department of International Agricultural Technology, Seoul National University, Pyeongchang, South Korea; ^3^ Department of Agricultural Biotechnology, Seoul National University, Seoul, South Korea; ^4^ Integrated Major in Global Smart Farm, Seoul National University, Seoul, South Korea

**Keywords:** cucumber mosaic virus, vector attraction, volatile cue, virus transmission, *Myzus persicae*

## Abstract

Plant viruses are obligate intracellular pathogens, and most depend on insect vectors for transmission between plants. Viral infection causes various physiological and metabolic changes in host traits, which subsequently influence the behavior and fitness of the insect vectors. Cucumber mosaic virus (CMV), one of the most widespread pathogens in pepper (*Capsicum annuum* L.), is transmitted by aphid vectors in a non-persistent manner. Here, we examined whether CMV infection in pepper affects the behavior of aphid vectors (*Myzus persicae* and *Aphis glycines*) in pepper. Aphid preference test revealed that significantly more aphids were attracted to CMV-infected pepper plants than to healthy plants. Comparative transcriptome analysis revealed a significant activation of the ethylene biosynthesis pathway in CMV-infected pepper plants. Indeed, gas chromatography analysis demonstrated that ethylene emission was significantly increased by CMV infection in pepper plants. Elevated ethylene emission in ethephon-treated healthy pepper increased their attractiveness to aphids. In contrast, aphid preference decreased after chemical inhibition of ethylene biosynthesis in CMV-infected pepper plants. Our results suggest that the ethylene emitted by CMV infection is a volatile cue that regulates the attractiveness of pepper plants to *M. persicae* and *A. glycines*.

## Introduction

Infection by pathogens induces a variety of physiological and metabolic changes in host plants, and these changes can subsequently influence the behavior and fitness of herbivorous insects that feed on these plants. Accumulating evidence suggests that pathogens can induce the release of volatile organic compounds (VOCs) from infected plants in ways that can either attract or repel insect herbivores (Eigenbrode et al., 2002; De Vos and Jander, 2010; Mauck et al., 2010; Mauck et al., 2014; Tungadi et al., 2017; Bak et al., 2019). Consequently, these host responses can strongly affect the transmission rates of insect-borne pathogens, with significant implications for epidemiology and agricultural ecology of insect-vectored diseases (Mauck et al., 2010; Mauck et al., 2014; Bak et al., 2019; Safari et al., 2019; Carr et al., 2020). However, little is still known about the biochemical and molecular mechanisms that mediate these interactions among pathogens, host plants, and insect herbivores.

Plant viruses are obligate intracellular pathogens that cause dramatic reprogramming of the transcriptome of host cells. This not only induces defense responses, but also disrupts normal plant development, physiology, and metabolism (Seo et al., 2018; Han et al., 2021). In nature, most plant viruses are transmitted between plants by insect vectors. Therefore, altering host plant traits in a manner that causes plants to attract insect vectors may be beneficial to the virus. Indeed, physiological and metabolic changes in virus-infected host plants have been shown to influence plant-insect vector interactions in ways that can increase virus transmission (Mauck et al., 2014; Tungadi et al., 2017; Bak et al., 2019). For example, changes in plant nutritional quality for vectors can enhance their performance and population growth (Mauck et al., 2014; Shikano et al., 2017). Viral infection can also increase the emission of plant VOCs that mediate vector attraction to plants (Mauck et al., 2010; Bak et al., 2019).

Aphid-transmitted potyviruses have been shown to enhance aphid performance on infected plants (Casteel et al., 2014; Adachi et al., 2018). Aphid vectors prefer to settle on host plants infected with potyviruses such as turnip mosaic virus and potato virus Y (PVY) and reproduce in larger numbers on infected host plants than on uninfected plants (Casteel et al., 2014; Bak et al., 2019). Potential mechanisms suggested for the enhanced aphid performance on potyvirus-infected plants include improvement of plant nutritional quality for aphids and suppression of plant defenses against aphids (Casteel et al., 2014; Adachi et al., 2018). Recently, PVY infection-induced ethylene signaling has been shown to be associated with aphid attraction and settlement on potato plants (Bak et al., 2019).

Cucumber mosaic virus (CMV; the type member of the genus *Cucumovirus*) is one of the most successful RNA viruses in terms of dispersion (Roossinck, 2002; Heo et al., 2020). CMV is a tripartite icosahedral virus transmitted by more than 70 species of aphids (Palukaitis and Garcia-Arenal, 2003). Aphids are phloem-feeding insects that transmit CMV in a non-persistent manner; therefore, CMV particles are retained in the aphid stylet for relatively short periods (a few minutes to a few hours) (Ng and Falk, 2006). In infected plants, CMV causes severe physiological and metabolic changes with diverse symptoms, including mosaic, stunting, leaf malformation, and chlorosis. Interestingly, CMV-infected plants show elevated emission of the VOC blend, which increases their attractiveness to aphid vectors (Mauck et al., 2010; Mauck et al., 2014). However, the specific VOC(s) responsible for manipulating aphid behavior have not been identified.

Pepper (*Capsicum annuum* L.) is an economically important vegetable crop worldwide, and CMV is one of the most widespread pathogens in this plant species (Kim et al., 2014a; Heo et al., 2020). Molecular investigations of CMV-pepper-aphid interactions can improve our understanding of the transmission ecology of CMV, which is non-persistently vectored by aphids in pepper. In the present study, we performed comparative transcriptome analysis to gain a molecular understanding of the physiological and metabolic changes in pepper plants in response to infection with CMV. Based on this approach, we observed a significant activation of the ethylene biosynthesis pathway in CMV-infected pepper plants. Ethylene is a volatile hormone associated with the susceptibility of various plant species to aphid infestation (Mantelin et al., 2009; Louis et al., 2015). Therefore, in this study, we also examined whether ethylene functions as a volatile cue mediating the attraction of aphid vectors to CMV-infected pepper plants.

## Materials and methods

### Biological materials

*Nicotiana benthamiana*, pepper (*Capsicum annuum* L. cv. Sinhong), and soybean (*Glycine max* cv. Lee 74) plants were grown in an insect-free growth chamber with a cycle of 16 h of light at 26°C and 8 h of darkness at 24°C. Full-length infectious cDNA clones of the CMV GTN strain (CMV-GTN) were used as viral sources (Heo et al., 2020). Infectious cDNA clones of CMV-GTN were inoculated into leaves of two-weeks-old *N. benthamiana* plants by agroinfiltration, as previously described (Seo et al., 2009; Heo et al., 2020). Crude sap prepared from the upper symptomatic leaves of *N. benthamiana* infected with CMV-GTN was used for mechanical inoculation of two-weeks-old pepper plants (Heo et al., 2020). Non-viruliferous clones of a pepper-adapted strain of the aphid *Myzus persicae* and a soybean-adapted strain of the aphid *Aphis glycines* were reared on pepper plants (cv. Sinhong) and soybean plants (cv. Lee 74), respectively, in a growth chamber with a cycle of 16 h of light at 26°C and 8 h of darkness at 24°C.

### Aphid preference test

**Figure 1:**
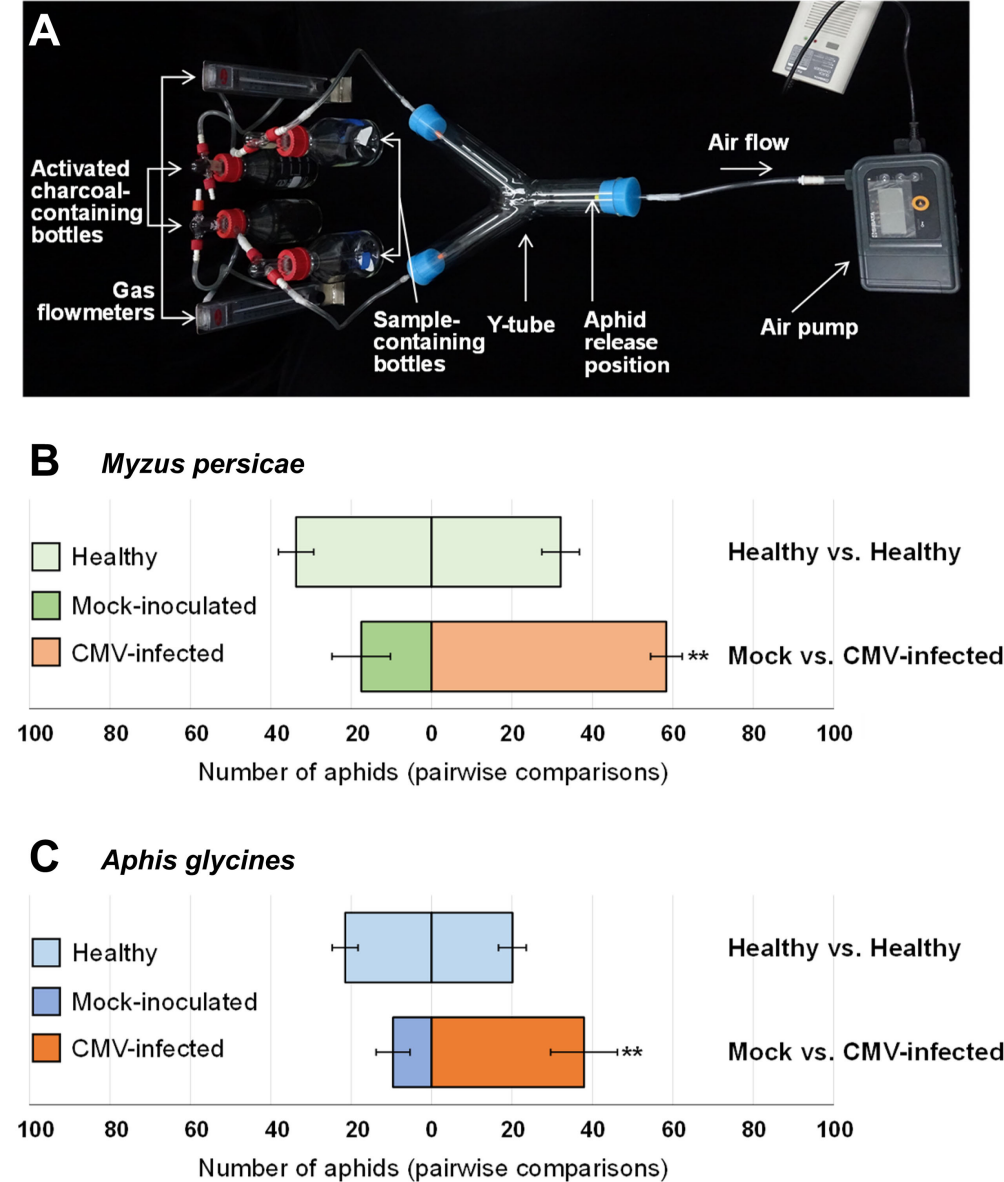
Aphid attraction to volatiles from healthy, mock-inoculated, or cucumber mosaic virus (CMV)-infected plants. **(A)** The glass Y-tube olfactometer used in this study for aphid preference tests. Pairwise preference tests for *Myzus persicae* (a species that colonizes pepper) **(B)** and *Aphis glycines* (a species that does not colonizes pepper) **(C)**. In pairwise aphid preference tests, pepper plants were treated as follows: healthy vs. healthy; mock-inoculated vs. CMV-infected. Pairs involved in each preference test are shown horizontally. Each pairwise test was repeated eight times, and data were averaged and are presented as the mean ± SD. Significant differences were analyzed using paired Student’s *t*-test (**P < 0.01).

Aphid preference for plant volatile cues was tested using a glass Y-tube olfactometer (diameter, 50 mm; length, 36 cm; **Figure 1A**) as described previously (Da Costa et al., 2011) with minor modifications. In brief, incoming air was filtered through activated charcoal and passed through two sample-containing chambers: one chamber contained a control sample, and the other contained a test sample. From each chamber, the air flowed into the respective arms of the Y-tube, and through the main tube of the olfactometer. Airflow through the olfactometer was maintained at 3.5 l/min using a gas flowmeter (RMB- 50D-SSV, Dwyer, USA). For each pairwise test, 100 wingless morphs of *M. persicae* or *A. glycines* were placed at the end of the main tube of the olfactometer and allowed to choose between two pepper samples. Tests were performed in the dark to eliminate any visual cues. The number of aphids that made a choice was counted at 3, 4, and 5 h after they were released, and the average was determined for each treatment in each test. Pairwise tests were performed to evaluate aphid preference for pepper plants treated as follows: (I) healthy vs. healthy, (II) mock-inoculated (mechanically inoculated with inoculation buffer) vs. CMV-infected, (III) healthy and mock-treated (water-sprayed) vs. healthy and ethephon-treated, (IV) CMV-infected and mock-treated vs. CMV-infected and aminoethoxyvinylglycine (AVG)-treated. Each pairwise test was repeated eight times, and the data were averaged.

### Sample preparation, library construction, and RNA sequencing

Fourteen days post-inoculation (dpi), symptomatic upper leaves were obtained from nine individual pepper plants infected with CMV-GTN. Leaf samples from three individual plants were pooled for RNA extraction. Thus, three biological replicates of RNA samples were obtained for each experimental group. Similarly, leaf samples from healthy plants were used as negative controls. Total RNA was isolated using PureLink^®^ RNA Mini Kit (Ambion, USA) and used for library construction with the Illumina TruSeq RNA Sample Preparation Kit v2 (Illumina, Inc., USA). Nine libraries were generated and quantified with the KAPA library quantification kit (Kapa Biosystems, USA). Sequencing was performed using an Illumina HiSeq2000 sequencer (Illumina, Inc., USA) by TheragenEtex Inc. (South Korea).

### Processing of RNA-seq data

RNA-seq data were analyzed as described previously (Han et al., 2021). Raw sequence reads were filtered using the Illumina pipeline and mapped to the reference transcripts of *C.* annuum cv. CM334 (Kim et al., 2014b) retrieved from Pepper Genome Platform (http://peppergenome.snu.ac.kr/). We used the RNA-seq mapping algorithm implemented in the bowtie2 software (v2.1.0) (Langmead et al., 2009) and allowed all alignments with a maximum of two mismatches. The raw data were deposited in the NCBI SRA database with BioProject accession numbers PRJNA751625 and PRJNA855293. To eliminate bias caused by variations in sequencing depth, the number of mapped clean reads for each gene was counted and normalized using the DESeq package in R software (Anders and Huber, 2010). Differentially expressed genes (DEGs) were identified based on by a ≥ two-fold change in read coverage and a false discovery rate (FDR) ≤ 0.01 in a binomial test. The FDR was applied to identify the threshold *p*-value for multiple tests and calculated using DESeq. Correlation analysis and hierarchical clustering were performed to categorize the genes according to their expression patterns using the AMAP library in R (Lucas, 2014).

### Gene enrichment analysis

Gene ontology (GO) analysis was used for functional annotation of the DEGs based on protein sequence similarity (e-value ≤ 1e-10) in the GO database (Ashburner et al., 2000). The in-house scripts of SEEDERS Inc. (Daejeon, South Korea) were used to count the number of DEGs assigned to each GO term. The PANTHER overrepresentation test was used to analyze GO term enrichment (Mi et al., 2013). The biological processes associated with enriched GO terms were visualized using the web-based REVIGO software (Supek et al., 2011), and the data were plotted in R. Functional enrichment analysis was performed to assign biological relevance to the gene network modules using agriGO v2.0 (Tian et al., 2017). The Kyoto Encyclopedia of Genes and Genomes (KEGG) pathway enrichment analysis was based on sequence similarity (e-value ≤ 1e-10; identity ≥ 90) between pepper proteins in the KEGG database (Kanehisa and Goto, 2000).

### Quantitative real-time polymerase chain reaction validation of RNA-seq data

To validate the RNA-seq results, the mRNA expression of the following genes was measured using qRT-PCR: *ripening-related protein grip22* (CA.PGAv.1.6.scaffold631.48), *glycine-rich protein* (CA.PGAv.1.6.scaffold1405.6), *AP2/ERF domain-containing transcription factor* (CA.PGAv.1.6.scaffold291.8), *basic-region leucine zipper transcription factor* (CA.PGAv.1.6.scaffold588.80), *n-methyltransferase 1-like* (CA.PGAv.1.6.scaffold423.30), *ACC oxidase-4-like* (CA.PGAv.1.6.scaffold784.1), *ACC oxidase-1-like* (CA.PGAv.1.6.scaffold793.14), *ACC synthase-2-like* (CA.PGAv.1.6.scaffold630.30), *ABC transporter B family member 11* (CA.PGAv.1.6.scaffold484.97), and *glycine-rich protein 5-like* (CA.PGAv.1.6.scaffold688.1). The *ubiquitin2* gene (CA.PGAv.1.6.scaffold337.91) was used as an internal reference for the qRT-PCR experiments, as this gene showed stable expression pattern in the RNA sequencing results. The same RNA preparations (DNase-treated) used for RNA-seq were used for cDNA synthesis using a Superscript III kit (Invitrogen, USA) with oligo-dT primers. The resulting cDNA was analyzed using SYBR Green-based qRT-PCR with specific primers (listed in **Supplementary Table S1**) in an iCycler iQ5 qRT-PCR detection system (Bio-Rad, USA). Three biological and three technical replicates were used per sample. The qRT-PCR results were normalized with the reference gene *ubiquitin2*, expressed as log2 fold changes in CMV-infected samples relative to healthy samples, and compared with log2 fold change data acquired from the RNA-seq.

### Ethylene analysis

Gas chromatography (GC) analysis was performed to measure ethylene production by pepper plants as described previously (Hou et al., 2018; Heo et al., 2020), with minor modifications. Briefly, the upper leaves were collected from three plants, and samples weighing 5 g were placed in 400-ml glass jars for 5 h at 26°C in the dark. A 1 ml air sample of the jar headspace was collected and analyzed using a GC system equipped with a hydrogen flame ionization detector and an activated alumina column (Agilent Technologies, USA). The samples were compared with a standard of known concentration. Each experiment was repeated three times, and the data were averaged. Ethylene analysis was performed for four-weeks-old pepper plants treated as follows: (I) healthy, (II) CMV-infected, (III) healthy and mock-treated (water-sprayed), (IV) healthy and ethephon-treated, (V) CMV-infected and mock-treated, and (VI) CMV-infected and AVG-treated.

### Chemical treatment

Pepper plants were treated with ethephon (Sigma-Aldrich Korea, Korea), which promotes ethylene release, or AVG (Sigma-Aldrich Korea, Korea), which inhibits ethylene biosynthesis. The treatments were performed as described previously (Bak et al., 2019), with minor modifications. To increase ethylene emission by plants, each four-weeks-old healthy pepper plant was sprayed homogeneously with 5 ml of 0.1 mg/l ethephon. Ethephon-treated plants were used in aphid preference tests and for ethylene analysis 2 h after the treatment. To inhibit ethylene release, 5 ml of 0.25 mM AVG was sprayed homogeneously on each four-weeks-old pepper plants inoculated with CMV-GTN 2 weeks previously. AVG-treated plants were used in aphid preference tests and for ethylene analysis 24 h after the treatment. Control plants (mock-treated) were sprayed with distilled water.

## Results

### Aphid vectors are more attracted to CMV-infected pepper plants than healthy plants

To examine whether CMV infection affects the attractiveness of host plants to aphid vectors, we performed pairwise preference tests using a Y-tube olfactometer. The tests were performed in the dark so that aphid vectors were exposed to volatile cues in the absence of visual cues. Significantly greater numbers of both *M. persicae* (a species that colonizes pepper) and *A. glycines* (a species that does not colonize pepper) preferred CMV-infected pepper plants instead of mock-inoculated plants (**Figure 1**). However, neither *M. persicae* nor *A. glycines* showed a preference between uninfected pepper plants (**Figure 1**). These results demonstrated that CMV infection in pepper plants resulted in increased release of volatile compounds to attract both colonizing and non-colonizing aphid vectors.

### Transcriptome of CMV-infected pepper

**Figure 2:**
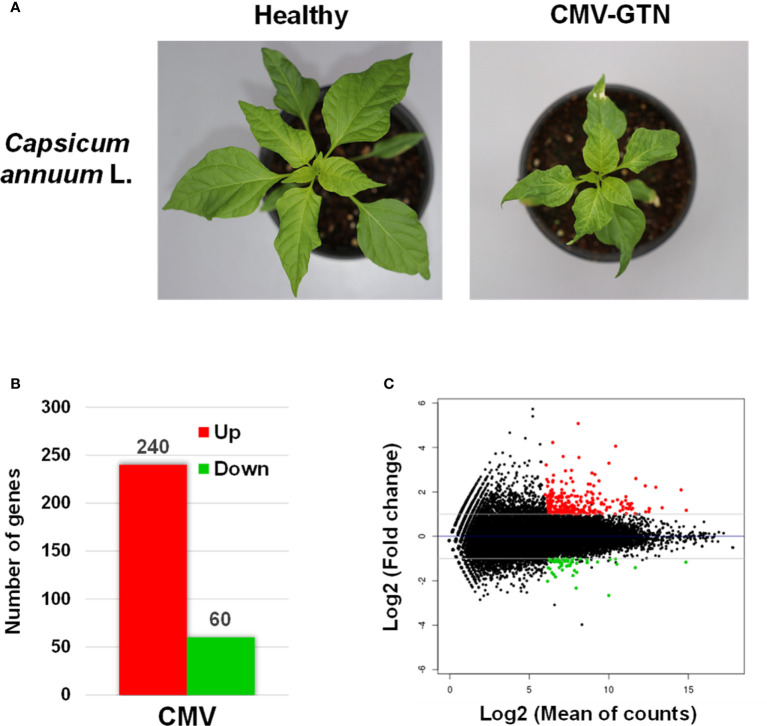
Transcriptomic reprogramming of pepper upon infection with CMV. **(A)** Phenotypes of healthy and CMV-infected pepper plants (*Capsicum annuum* L.) subjected to transcriptome analysis. Plants infected with CMV-GTN exhibited mosaic, severe leaf size reduction, and stunted growth. **(B)** Number of differentially expressed genes (DEGs) identified using RNA-seq of CMV-infected pepper plants. The DEGs were identified by comparing the transcriptomes of CMV-infected and healthy pepper plants (two-fold change in expression with a false discovery rate of ≤ 0.01). Red and green bar indicates the number of up- and downregulated DEGs, respectively. **(C)** MA-plot of RNA seq results. Each dot represents one gene. The normalized mean of read counts is shown on the x axis, and the log2 fold change of normalized counts is shown on the y axis. Up- and downregulated DEGs are represented by red and green dots, respectively.

We next examined CMV-induced transcriptomic changes in pepper plants to identify host genes associated with the increased attractiveness to aphid vectors. Samples of total RNA isolated from healthy and CMV-infected pepper plants (**Figure 2A**) were used for cDNA library construction. Six cDNA libraries (three for each sample) were constructed and sequenced using an Illumina RNA-seq system. Each library yielded approximately 81–89 million clean paired-end reads (**Supplementary Table S2**) that were mapped to 35,884 reference transcripts of *C. annuum* cv. CM334 (Kim et al., 2014b). Approximately 92% of the nucleotides were mapped; the nucleotide coverage for healthy and CMV-infected samples was 175- and 192.33-fold, respectively (**Supplementary Table S2**).

### DEGs associated with response to CMV infection

To normalize the expression levels of the mapped genes, we used the fragments per kilobase of exon per million fragments mapped (FPKM) value. The FPKM values of the CMV-infected samples were compared with those of the healthy samples, and DEGs were identified based on a two-fold change in expression with FDR ≤ 0.01. We identified 300 DEGs (240 upregulated and 60 downregulated genes) in response to infection with CMV-GTN (**Figure 2B**, **Tables 1**, **2** and **Supplementary Table S3**). The top 30 DEGs upregulated upon infection with CMV included a few ethylene-responsive genes (e.g. CA.PGAv.1.6.scaffold631.48 and CA.PGAv.1.6.scaffold291.8) and various defense response-related genes (**Table 1**). The latter group included genes encoding proteins in the chitinase family (CA.PGAv.1.6.scaffold939.24, CA.PGAv.1.6.scaffold890.65, CA.PGAv.1.6.scaffold1306.1, CA.PGAv.1.6.scaffold13678.1, CA.PGAv.1.6.scaffold1611.1, CA.PGAv.1.6.scaffold1306.2, and CA.PGAv.1.6.scaffold1306.3) and receptor-like protein kinase genes (CA.PGAv.1.6.scaffold674.24, CA.PGAv.1.6.scaffold1110.29, and CA.PGAv.1.6.scaffold387.5) (**Table 1**). It was very Interesting that various chitinase family protein genes and ethylene-responsive genes were dramatically upregulated after infection with CMV (**Table 1**). To examine changes in global gene expression in CMV-infected samples, we visualized the magnitude distribution of DEG counts using an MA plot (**Figure 2C**). In general, fold changes were higher for upregulated DEGs than for down-regulated DEGs.

**Table 1:** Top 30 upregulated DEGs in response to infection with CMV.

Gene ID	Seq. description	log2 fold change	*Arabidopsis*homolog
CA.PGAv.1.6.scaffold631.48	Ripening-related protein grip22	5.08	NA
CA.PGAv.1.6.scaffold674.24	Cysteine-rich receptor-like protein kinase 25	4.22	AT4G05200.1
CA.PGAv.1.6.scaffold1405.6	Glycine-rich protein	4.06	NA
CA.PGAv.1.6.scaffold291.8	AP2/ERF domain-containing transcription factor	3.59	AT5G13330.1
CA.PGAv.1.6.scaffold889.4	tRNA/rRNA methyltransferase family protein	3.55	AT4G15520.1
CA.PGAv.1.6.scaffold939.24	Chitinase family protein (PR-3)	3.29	AT2G43600.1
CA.PGAv.1.6.scaffold575.23	Fatty acid hydroxylase superfamily	3.21	AT1G02190.2
CA.PGAv.1.6.scaffold890.65	Chitinase family protein (PR-3)	2.97	AT3G12500.1
CA.PGAv.1.6.scaffold21.10	Alkaline alpha-galactosidase seed imbibition protein	2.95	AT1G55740.1
CA.PGAv.1.6.scaffold588.80	Basic-region leucine zipper transcription factor	2.85	AT2G16770.1
CA.PGAv.1.6.scaffold1306.1	Chitinase family protein (PR-3)	2.79	AT3G12500.1
CA.PGAv.1.6.scaffold1110.29	G-type lectin S-receptor-like serine/threonine-protein kinase	2.75	AT4G27290.1
CA.PGAv.1.6.scaffold1134.16	Protein of unknown function	2.62	NA
CA.PGAv.1.6.scaffold13678.1	Chitinase family protein (PR-3)	2.60	AT3G12500.1
CA.PGAv.1.6.scaffold889.10	Pyruvate orthophosphate dikinase	2.56	AT4G15530.6
CA.PGAv.1.6.scaffold370.26	Responsive to dehydration 21B-like	2.51	AT5G43060.1
CA.PGAv.1.6.scaffold423.30	n-methyltransferase 1-like	2.50	AT3G18000.1
CA.PGAv.1.6.scaffold254.2	SWEET sucrose efflux transporter family protein	2.47	AT5G23660.1
CA.PGAv.1.6.scaffold1611.1	Chitinase family protein (PR-3)	2.46	AT3G12500.1
CA.PGAv.1.6.scaffold241.41	MYB transcription factor 59-like	2.45	AT5G59780.3
CA.PGAv.1.6.scaffold387.5	LRR protein kinase family protein	2.45	AT3G42880.1
CA.PGAv.1.6.scaffold129.43	PRLI-interacting factor	2.37	AT5G19900.1
CA.PGAv.1.6.scaffold1716.3	Protein of unknown function	2.35	AT2G42670.2
CA.PGAv.1.6.scaffold464.22	Cytochrome P450	2.35	AT3G14690.1
CA.PGAv.1.6.scaffold1268.8	Ferritin 2-like	2.27	AT3G11050.1
CA.PGAv.1.6.scaffold1306.2	Chitinase family protein (PR-3)	2.27	AT3G12500.1
CA.PGAv.1.6.scaffold23.1	NAC domain-containing protein 36-like	2.26	AT2G17040.1
CA.PGAv.1.6.scaffold1306.3	Chitinase family protein (PR-3)	2.21	AT3G12500.1
CA.PGAv.1.6.scaffold551.8	Glycine-rich cell wall structural protein	2.20	AT4G30460.1
CA.PGAv.1.6.scaffold1058.13	phosphatidylinositol transfer family protein	2.20	AT4G36490.1

NA, not available.

**Table 2:** Top 20 downregulated DEGs in response to infection with CMV.

Gene ID	Seq. description	log2 fold change	*Arabidopsis*homolog
CA.PGAv.1.6.scaffold484.97	Diacylglycerol O-acyltransferase 3	-2.66	NA
CA.PGAv.1.6.scaffold5.23	Glycine-rich protein 5-like	-2.33	NA
CA.PGAv.1.6.scaffold303.33	O-methyltransferase family protein	-2.02	AT5G54160.1
CA.PGAv.1.6.scaffold702.9	Alpha/beta-Hydrolases superfamily protein	-1.83	AT4G18550.1
CA.PGAv.1.6.scaffold688.1	Glycine-rich protein 5-like	-1.76	NA
CA.PGAv.1.6.scaffold1217.13	Putative xyloglucan galactosyltransferase KATAMARI1-like	-1.72	NA
CA.PGAv.1.6.scaffold410.33	RmlC-like cupins superfamily protein	-1.64	AT3G05950.1
CA.PGAv.1.6.scaffold132.22	Terpene synthase 14-like	-1.62	AT1G61680.1
CA.PGAv.1.6.scaffold1174.1	Terpene synthase 14-like	-1.54	AT1G61680.1
CA.PGAv.1.6.scaffold1559.2	Fatty acid desaturase 3-like	-1.51	AT2G29980.1
CA.PGAv.1.6.scaffold1030.35	LRR protein kinase family protein	-1.47	AT5G43020.1
CA.PGAv.1.6.scaffold1129.9	ATP binding microtubule motor family protein	-1.42	AT1G18370.1
CA.PGAv.1.6.scaffold589.2	GTP binding Elongation factor Tu family protein	-1.41	AT5G60390.1
CA.PGAv.1.6.scaffold861.41	Proteinase inhibitor type-2	-1.41	NA
CA.PGAv.1.6.scaffold569.23	Cytochrome P450	-1.34	AT1G11600.1
CA.PGAv.1.6.scaffold771.42	Subtilase family protein	-1.33	AT1G04110.1
CA.PGAv.1.6.scaffold322.14	Serine carboxypeptidase-like	-1.32	AT3G63470.1
CA.PGAv.1.6.scaffold572.61	pEARLI1-like lipid transfer protein	-1.32	NA
CA.PGAv.1.6.scaffold916.12	LRR-like protein kinase family protein	-1.32	AT5G62230.1
CA.PGAv.1.6.scaffold198.27	Protein of unknown function	-1.27	NA

NA, not available.

### GO term enrichment for identified DEGs

**Figure 3:**
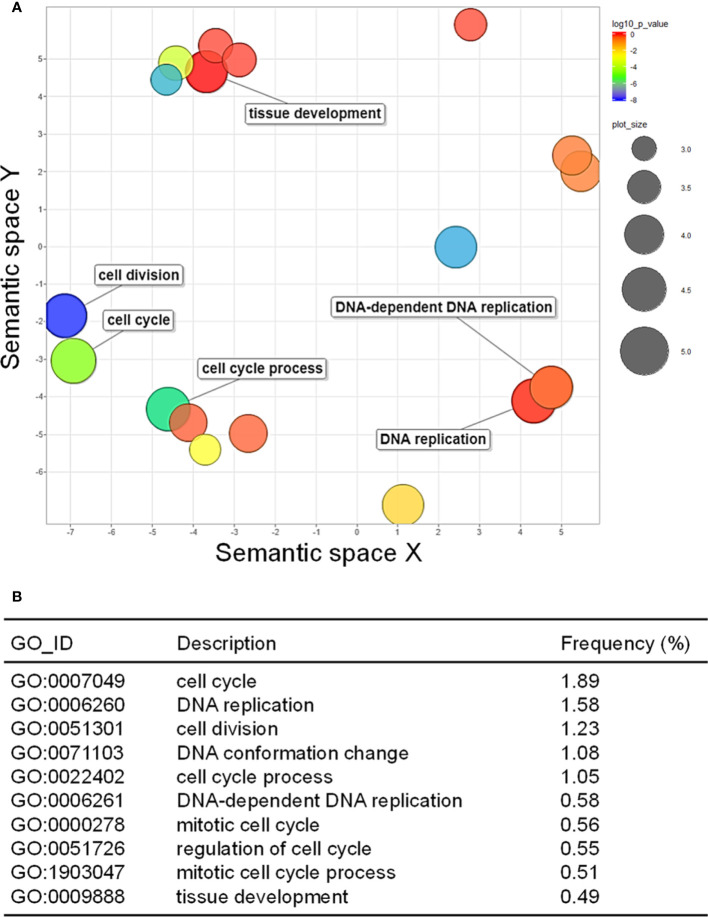
Gene ontology (GO) enrichment of downregulated differentially expressed genes (DEGs). **(A)** GO enrichment in the biological process category, as visualized using REVIGO. The scatter plot shows the significance of GO terms in a two-dimensional space. Multi-dimensional scaling was applied to a matrix of GO terms to calculate semantic similarities. Colors indicate the *P*-value for the false discovery rate, and circle sizes represent the frequency of the GO term. **(B)** Top 10 GO terms enriched for the downregulated DEGs in response to CMV infection.

**Figure 4:**
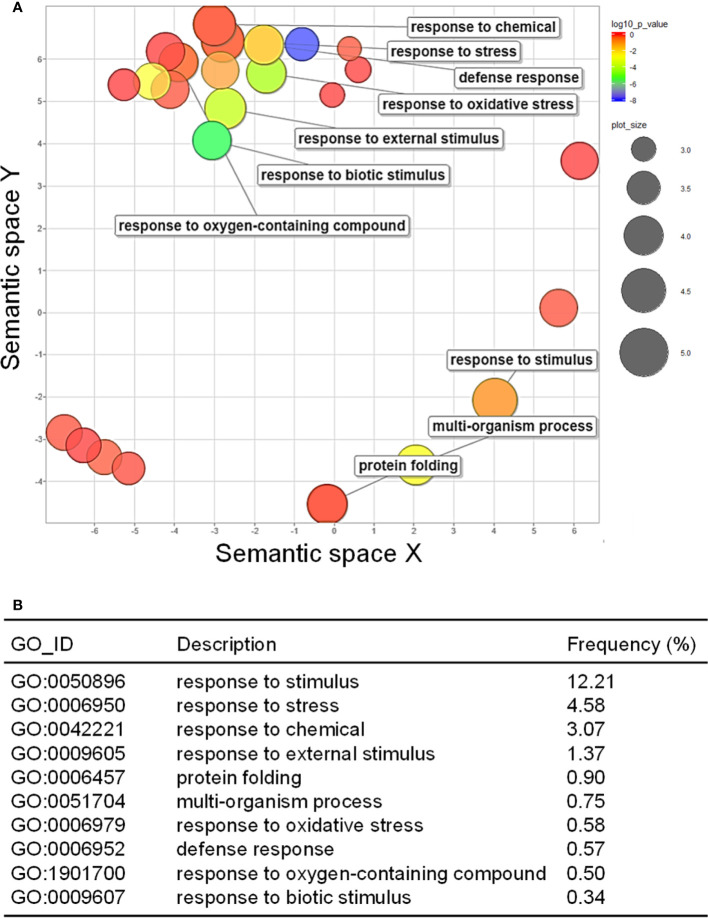
GO enrichment of upregulated DEGs. **(A)** GO enrichment in the biological process category, as visualized using REVIGO. The scatter plot shows the significance of GO terms in a two-dimensional space. Multi-dimensional scaling was applied to a matrix of GO terms to calculate semantic similarities. Colors indicate the *P*-value for the false discovery rate, and circle sizes represent the frequency of the GO term. **(B)** Top 10 GO terms enriched for the upregulated DEGs in response to CMV infection.

GO analysis was performed to examine how the up- and downregulated DEGs were associated with CMV infection-induced metabolic and physiological changes. A total of 98 GO terms (59 and 39 GO terms for up- and downregulated DEGs, respectively) were significantly enriched after infection with CMV (**Supplementary Table S4**). The enriched GO terms associated with the downregulated DEGs were related to regulation of cell division and included the following: cell cycle (GO:0007049), DNA replication (GO:0006260), cell division (GO:0051301), DNA conformation change (GO:0071103), and cell cycle process (GO:0022402) (**Figures 3A, B**). CMV infection severely reduced leaf size and caused stunted growth in pepper plants (**Figure 2A**). The morphology of plant organs is intricately regulated by cell division and expansion (Meyerowitz, 1997). Therefore, the symptoms induced by CMV may be directly associated with the suppression of genes that regulate cell division. The GO terms associated with the upregulated DEGs included those related to cellular responses to various stimuli: response to stimulus (GO:0050896), response to stress (GO:0006950), response to chemical (GO:0042221), and response to external stimulus (GO:0009605) (**Figures 4A, B**). Hierarchical GO enrichment analysis using agriGO v2.0 revealed that the enriched GO terms were highly correlated in a network context (**Supplementary Figure S1**).

### KEGG pathways enriched by CMV infection

**Figure 5:**
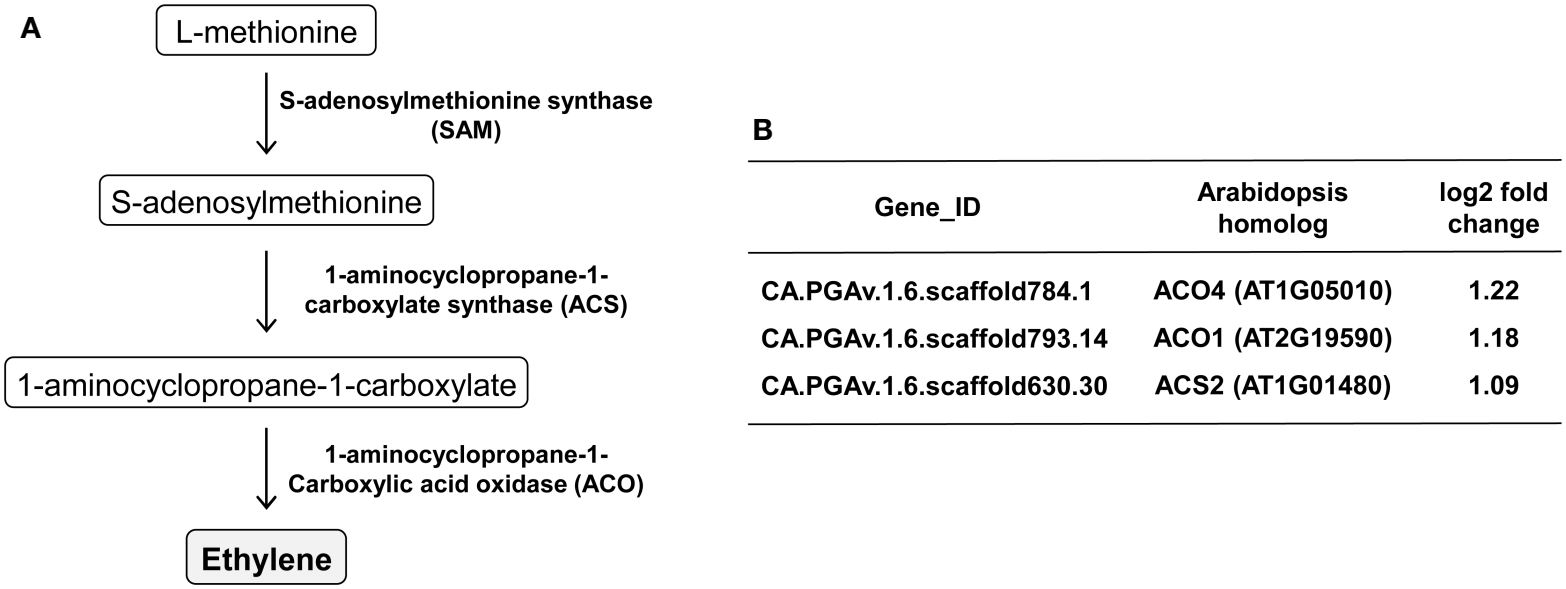
Regulation of genes involved in ethylene biosynthesis upon CMV infection in pepper. **(A)** The ethylene biosynthesis pathway and key enzymes. **(B)** Ethylene biosynthesis-associated genes upregulated in response to CMV infection. The changes in gene expression were calculated using a log scale of the FPKM data obtained using RNA-seq.

Next, KEGG pathway analysis was performed to examine the expression patterns of the DEGs in the associated pathways. KEGG analysis of the upregulated DEGs identified some highly ranked pathways, including plant hormone signal transduction (Pathway ID: cann04075), cysteine and methionine metabolism (Pathway ID: cann00270), and biosynthesis of secondary metabolites (Pathway ID: cann01110) (**Table 3**). It was interesting that CMV infection caused significant changes in important pathways related to metabolite biosynthesis, because we performed transcriptome analysis to identify the VOCs responsible for attracting aphids to plants. In particular, the cysteine and methionine metabolism pathway include the ethylene biosynthesis pathway, and we found that key genes involved in ethylene biosynthesis, including 1-aminocyclopropane-1-carboxylate (ACC) synthetase (ACS) and ACC oxidase (ACO), were significantly upregulated upon infection with CMV (**Figure 5**).

**Table 3:** Top 5 KEGG pathways enriched for DEGs up-regulated in response to CMV infection.

Pathway	Pathway ID	DEGs	*p*-value
Plant hormone signal transduction	cann04075	18	9.10E-07
Cysteine and methionine metabolism	cann00270	11	4.00E-06
Biosynthesis of secondary metabolites	cann01110	33	7.20E-05
ABC transporters	cann02010	4	6.80E-03
Diterpenoid biosynthesis	cann00904	3	5.00E-02

### qRT-PCR validation of RNA-seq data

To validate the RNA-seq results, the expression of 10 representative DEGs was assessed by qRT-PCR using the same RNA preparations used for RNA-seq. The expression of these target genes was normalized to that of *ubiquitin2* (CA.PGAv.1.6.scaffold337.91), which was used as an internal control. The relative expression of each gene was calculated and compared with the corresponding RNA-seq data. The qRT-PCR results of all evaluated genes were generally consistent with their respective RNA-seq results (**Supplementary Table S5**). This demonstrated the accuracy of gene expression data obtained using RNA-seq in this study.

### CMV infection increases ethylene emission in pepper

**Figure 6:**
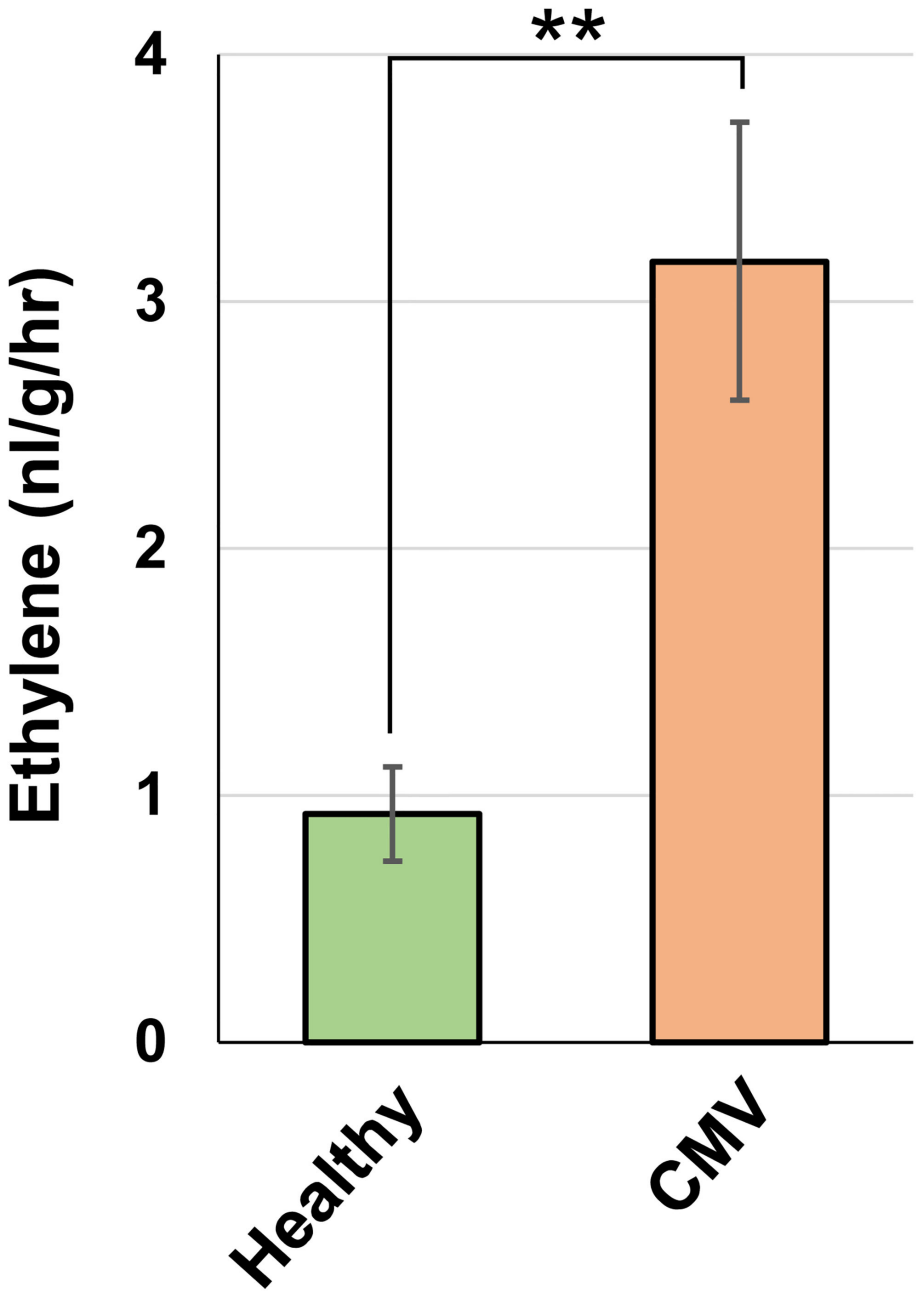
Effect of CMV infection on ethylene emission in pepper. Ethylene emission by upper uninoculated leaves of healthy and CMV-infected plants, as measured using gas chromatography. The mean ± SD values from three independent experiments are shown, and each column represents one group with nine plants. Significant differences were determined using paired Student’s *t*-test (**P < 0.01).

Our transcriptomic analysis revealed that CMV infection activated the ethylene biosynthesis pathway in pepper. Therefore, we investigated whether ethylene production is indeed enhanced in CMV-infected pepper plants. GC analysis demonstrated that ethylene emission was considerably higher in CMV-infected leaves than in healthy leaves (**Figure 6**). This implied that CMV infection increased ethylene production in pepper, which was consistent with the transcriptional regulation of the ethylene biosynthesis pathway.

### Ethylene is a volatile chemical cue mediating aphid attraction

**Figure 7:**
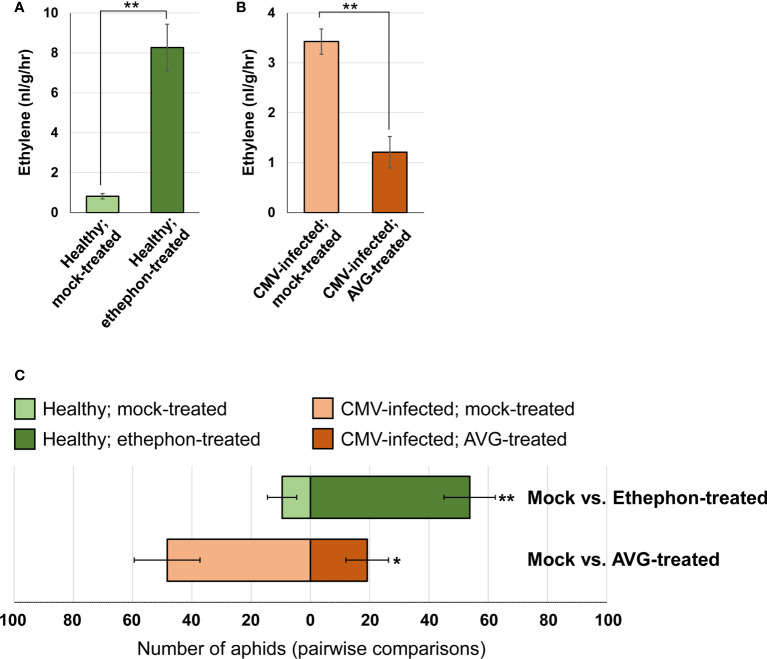
Effect of changes in ethylene emission on aphid attraction to pepper plants. **(A)** Ethylene emission by healthy pepper plants treated with mock or ethephon, as measured using gas chromatography. **(B)** Ethylene emission by CMV-infected pepper plants treated with mock or AVG, as measured using gas chromatography. The mean ± SD values from three independent experiments are shown, and each column represents one group with nine plants. Significant differences were determined using paired Student’s *t*-test (**P < 0.01). **(C)** Pairwise aphid preference tests between pepper plants treated as follows: healthy and mock-treated vs. healthy and ethephon-treated; CMV-infected and mock-treated vs. CMV-infected and AVG-treated. Pairs involved in each preference test are shown horizontally. Each pairwise test was repeated eight times, and data were averaged and are presented as the mean ± SD. Significant differences were determined using paired Student’s *t*-test (*P < 0.05; **P < 0.01).

Ethylene is a volatile hormone, and its production was strongly upregulated in CMV-infected pepper plants. Therefore, we next investigated whether ethylene acts as a volatile cue to mediate aphid attraction to pepper plants. We utilized two chemical compounds: ethephon (a promoter of ethylene release) and AVG (an inhibitor of ethylene biosynthesis) (Bak et al., 2019). Ethephon treatment increased ethylene emission in healthy pepper plants (**Figure 7A**), and a greater number of *M. persicae* preferred ethephon-treated pepper plants over mock-treated plants (average 51.8 and 8.6, respectively; **Figure 7C**). Furthermore, AVG treatment inhibited ethylene emission in CMV-infected pepper plants (**Figure 7B**). The number of aphids that preferred AVG-treated pepper was less than half the number of those that preferred mock-treated pepper (average 20 and 46.3, respectively; **Figure 7C**). Therefore, our results suggested that ethylene emitted by pepper plants acts as a volatile cue responsible for attracting aphid vectors.

## Discussion

Despite being a non-persistently transmitted virus, CMV has evolved into one of the most successful plant viruses in terms of worldwide dispersion (Palukaitis and Garcia-Arenal, 2003; Heo et al., 2020). The successful transmission of a virus requires that virus-infected plants attract insect vectors and stimulate them to acquire virus particles. Following this, the vectors must migrate to neighboring plants to transmit the virus within a short period. This pattern of transmission has been demonstrated in interactions between aphid vectors and squash (*Cucurbita pepo*) plants infected with CMV. In this system, the aphid vectors are preferentially attracted to CMV-infected squash plants but migrate to nearby plants soon after initial feeding (Mauck et al., 2010; Mauck et al., 2014). In this regard, previous studies found that CMV infection causes the following physiological and biochemical changes in host plants: (1) reduced plant nutritional quality for vectors; (2) induction of plant defense responses against aphid feeding; and (3) elevated emission of plant VOCs (Mauck et al., 2010; Mauck et al., 2014). These findings suggest that elucidating the molecular mechanisms underlying these virus-induced phenotypic and physiological changes in host plants is essential for better understanding of the interactions between host plants and insect vectors in relation to viral transmission.

Consistent with the aforementioned observations in squash plants (Mauck et al., 2010), we demonstrated that CMV infection increased the attractiveness of pepper plants to both colonizing and non-colonizing aphid vectors (**Figure 1**). We employed comparative transcriptome analysis to explore the molecular basis of physiological and metabolic changes in pepper plants in response to infection with CMV. Based on this approach, we identified several host genes associated with the increased attractiveness of CMV-infected plants to aphid vectors. Our analysis of DEGs and GO terms showed that many upregulated DEGs were associated with cellular responses to various stimuli, including defense responses (**Figure 4**; **Supplementary Figure S1**; **Table 1**). KEGG pathway analysis further highlighted that several DEGs were associated with biosynthesis of metabolites, especially ethylene (**Figure 5**; **Table 3**). The upregulation of key genes involved in ethylene biosynthesis, including ACS (CA.PGAv.1.6.scaffold630.30) and ACO(CA.PGAv.1.6.scaffold784.1 and CA.PGAv.1.6.scaffold793.14), resulted in elevated ethylene production in CMV-infected plants, indicating that CMV infection increases ethylene emission in pepper (**Figure 6**).

Ethylene is an important plant hormone that regulates many aspects of plant development, and plays a key role in modulating plant responses to biotic and abiotic stresses (Van Loon et al., 2006; Huang et al., 2016). Accumulating evidence suggests that virus-induced ethylene signaling is associated with the alteration of host plant susceptibility (Louis et al., 2015; Han et al., 2021). A recent study reported that in pepper plants infected with broad bean wilt virus 2 (BBWV2), elevated ethylene production enhances the severity of disease symptoms (Han et al., 2021). In rice, infection with rice dwarf virus (RDV) caused a dramatic increase in ethylene production, resulting in increased susceptibility of rice plants to RDV (Zhao et al., 2017). Interestingly, RDV infection increased the host preference of its insect vector, the green rice leafhopper (Wang et al., 2018), although the role of RDV-induced ethylene emission in causing this change has not been examined. A recent study demonstrated that PVY-induced ethylene mediates aphid attraction to infected potato plants, thus affecting virus spread (Bak et al., 2019). Several studies have also implicated virus-induced VOCs in vector attraction to infected plants (Eigenbrode et al., 2002; Ngumbi et al., 2007; Mauck et al., 2010; Mauck et al., 2014; Tungadi et al., 2017; Bak et al., 2019).

Ethylene is a volatile hormone, and its emission levels were higher in pepper plants infected with CMV (**Figure 6**). In this study, we showed that the elevated levels of ethylene emitted by ethephon-treated uninfected pepper plants increased their attractiveness to aphids (**Figure 7**). In contrast, the inhibition of ethylene biosynthesis in AVG-treated CMV-infected pepper plants reduced their attractiveness to aphids (**Figure 7**). Taken together, these results suggest that CMV-induced changes in ethylene production alters the attractiveness of pepper plants to aphid vectors. However, we cannot rule out the possibility that CMV-induced ethylene signaling affects the overall production of VOCs in pepper plants, which may be important for aphid attraction as well.

Rapid dispersal of insect vectors after initial feeding is important for successful transmission of non-persistently transmitted viruses (Mauck et al., 2010). Aphids show a strong tendency to leave CMV-infected squash plants after initial feeding (Mauck et al., 2010; Mauck et al., 2014), suggesting that CMV infection induces gustatory cues that stimulate aphids to disperse soon after feeding. A previous study in tobacco plants demonstrated that CMV infection induced plant defense signaling and H_2_O_2_ production. The authors suggested that CMV-induced H_2_O_2_ may function as a gustatory cue to modify aphid feeding behavior in ways that enhance its virus acquisition and aphid dispersal (Guo et al., 2019). Our transcriptome analysis revealed that several DEGs upregulated in response to CMV infection in pepper were associated with plant defense responses. These DEGs included gene encoding receptor-like protein kinases and pathogenesis-related proteins as well as genes involved in hormone signaling pathways (**Table 1** and **Figure 4**). More interestingly, several genes encoding proteins in the chitinase family were also highly upregulated in CMV-infected pepper (**Table 1**). Chitin is a major component of the exoskeleton and alimentary canal of insects, whereas chitinases are hydrolytic enzymes that break down glycosidic bonds in chitin (Ding et al., 1998). Various insect chitinase genes have been used to generate transgenic plants resistant to insect pests (Ding et al., 1998; Schuler et al., 1998; Arakane and Muthukrishnan, 2010). Furthermore, the overexpression of plant chitinases enhanced the resistance of *Arabidopsis* and potato plants to aphids (Gatehouse et al., 1996; Zhong et al., 2021). Therefore, we suggest that the chitinase family proteins induced by CMV infection may affect plant resistance to aphids and act as a gustatory cue to stimulate post-feeding dispersal in insect vectors.

Vector-borne pathogens may have evolved to increase their transmission efficiency by altering host-vector-pathogen interactions to facilitate viral transmission (Mauck et al., 2016). Viral infection causes complex physiological and metabolic changes in host plants owing to transcriptomic reprogramming (Seo et al., 2018; Han et al., 2021). Our transcriptome analysis of CMV-pepper plant interactions revealed molecular genetic clues regarding the manipulated behaviors of aphid vectors feeding on CMV-infected pepper plants. CMV infection upregulated genes related to ethylene biosynthesis (**Figure 5**), and subsequent experiments (**Figure 7**) suggested that enhanced ethylene emission is responsible for aphid attraction in pepper. CMV infection also induced various genes encoding proteins in the chitinase family that may stimulate aphid dispersal (**Table 1**). Taken together, our findings improve our understanding of the molecular mechanisms underlying virus-induced manipulation of plant-insect interactions. Moreover, these results provide important insights into the evolutionary relationships among plant hosts, viruses, and insect vectors.

## Data availability statement

The datasets presented in this study can be found in online repositories. The names of the repository/repositories and accession number(s) can be found in the article/**Supplementary Material**.

## Author contributions

S-JK and J-KS designed the experiments and supervised the project; S-JK, S-JH, M-HK, S-YJ, and J-SC performed the experiments; S-JK, S-JH, and J-KS analyzed the data; S-JK, S-JH, and J-KS wrote and revised the manuscript. All authors contributed to the article and approved the submitted version.

## Funding

This research was supported in part by grants from Basic Science Research Program (NRF-2020R1I1A1A01072564) funded by the National Research Foundation of Korea and Agenda Program (PJ015308) funded by the Rural Development Administration of Korea.

## Conflict of interest

The authors declare that the research was conducted in the absence of any commercial or financial relationships that could be construed as a potential conflict of interest.

## Publisher’s note

All claims expressed in this article are solely those of the authors and do not necessarily represent those of their affiliated organizations, or those of the publisher, the editors and the reviewers. Any product that may be evaluated in this article, or claim that may be made by its manufacturer, is not guaranteed or endorsed by the publisher.

## Supplementary material

Supplementary Table 1 Primers used in this study for qRT-PCR.

Supplementary Table 2 Summary statistics of sequences obtained from RNA-seq of pepper plants infected with CMV.

Supplementary Table 3 DEGs in response to infection with CMV-GTN.

Supplementary Table 4 GO terms for DEGs in response to infection with CMV-GTN.

Supplementary Table 5 Validation of RNA sequencing data by qRT-PCR.

Supplementary Figure 1 Hierarchical tree graph of overrepresented GO terms for upregulated DEGs in response to CMV infection.
