# Supplementary material for: Ethylene emitted by viral pathogen-infected pepper (Capsicum annuum L.) plants is a volatile chemical cue that attracts aphid vectors
Source: Front Plant Sci. 2022 Sep 29;13:994314. doi: 10.3389/fpls.2022.994314 (PMC9559363; doi:10.3389/fpls.2022.994314)
Supplement: Supplementary Table 1 — Primers used in this study for qRT-PCR. [file Table_1.doc]

Supplementary Table S1. Primers used in this study for qRT-PCR.

| **Primer** | **Primer sequence (5’ to 3’)** | **Target gene** |
| --- | --- | --- |
| grip22-F | CCCTCCTACTCCAACCAATAC | ripening-related protein grip22  (CA.PGAv.1.6.scaffold631.48) |
| grip22-R | AACGTCCTGCAAACCATCC |
| GRP-F | GGGAAAAGCCACTGAATCAC | glycine-rich protein  (CA.PGAv.1.6.scaffold1405.6) |
| GRP-R | GACCAACCATCCATTGGAAC |
| ERF-F | TCAGGGAGCGGGAGAAGACAT | AP2/ERF domain-containing transcription factor  (CA.PGAv.1.6.scaffold291.8) |
| ERF-R | TGATGGGAATTATGCTGAGGTGAA |
| BLZ-F | CCGCCCGGTCCTGATAACTC | basic-region leucine zipper transcription factor  (CA.PGAv.1.6.scaffold588.80) |
| BLZ-R | GCAACCTCCGCTTCCAACACA |
| MT1-F | GTGTTGGAATTGGGTGCTGGTA | n-methyltransferase 1-like  (CA.PGAv.1.6.scaffold423.30) |
| MT1-R | ATATAGCCCCCAACTTTCAACCAT |
| ACO4-F | CACCATGCCCCAAGCCTGAT | ACC oxidase-4 like  (CA.PGAv.1.6.scaffold784.1) |
| ACO4-R | CATGCGAGTCCCGTTTGTTTGT |
| ACO1-F | GGCTCCTAATCCTCCCAGTCCA | ACC oxidase-1 like  (CA.PGAv.1.6.scaffold793.14) |
| ACO1-R | AGGCTGAGGACATGCTGGATAG |
| ACS2-F | CAGCCATTTGACGGGTTGTTG | ACC synthase-2 like  (CA.PGAv.1.6.scaffold630.30) |
| ACS2-R | GCACTACGGCAAGGAACAGGT |
| ABC11-F | GCTATACTAGACGAACTCCAAC | ABC transporter B family member 11  (CA.PGAv.1.6.scaffold484.97) |
| ABC11-R | CACATCCTCTAAACCAACTCC |
| GRP5-F | GTTCAGACCACAGCAGCAAGAG | glycine-rich protein 5-like  (CA.PGAv.1.6.scaffold688.1) |
| GFP5-R | CACCACCAGCACCAACACCAGT |
| UBI2-F | TACCCTTCACCTTGTCCTCC | ubiquitin2 |
| UBI2-R | GCCATCCTCCAACTGTTTTC |
