# Supplementary material for: Ethylene emitted by viral pathogen-infected pepper (Capsicum annuum L.) plants is a volatile chemical cue that attracts aphid vectors
Source: Front Plant Sci. 2022 Sep 29;13:994314. doi: 10.3389/fpls.2022.994314 (PMC9559363; doi:10.3389/fpls.2022.994314)
Supplement: Supplementary Table 2 — Summary statistics of sequences obtained from RNA-seq of pepper plants infected with CMV. [file Table_2.doc]

Supplementary Table S2. Summary statistics of sequences obtained from RNA-seq of pepper plants infected with CMV.

| Index | Healthy | CMV-infected |
| --- | --- | --- |
| No. of trimmed reads (%) | 80,988,234  (90.73%) | 89,158,100  (91.55%) |
| No. of mapped reads (%) | 75,175,224  (92.82%) | 82,327,921  (92.34%) |
| No. of mapped nucleotides (%) | 6,848,712,056  (92.81%) | 7,526,945,055  (92.55%) |
| Average coverage | 175.00 | 192.33 |
