# Supplementary material for: Ethylene emitted by viral pathogen-infected pepper (Capsicum annuum L.) plants is a volatile chemical cue that attracts aphid vectors
Source: Front Plant Sci. 2022 Sep 29;13:994314. doi: 10.3389/fpls.2022.994314 (PMC9559363; doi:10.3389/fpls.2022.994314)
Supplement: Supplementary Table 5 — Validation of RNA sequencing data by qRT-PCR. [file Table_5.doc]

Supplementary Table S5. Validation of RNA sequencing data by qRT-PCR

| **Gene** | **Seq. Description** | **Log2-fold change by RNA sequencing** | **Log2-fold change by qRT-PCR** |
| --- | --- | --- | --- |
| CA.PGAv.1.6.scaffold631.48 | ripening-related protein grip22 | 5.08 | 4.53 |
| CA.PGAv.1.6.scaffold1405.6 | glycine-rich protein | 4.06 | 4.35 |
| CA.PGAv.1.6.scaffold291.8 | AP2/ERF domain-containing transcription factor | 3.59 | 3.84 |
| CA.PGAv.1.6.scaffold588.80 | basic-region leucine zipper transcription factor | 2.85 | 2.19 |
| CA.PGAv.1.6.scaffold423.30 | n-methyltransferase 1-like | 2.50 | 2.36 |
| CA.PGAv.1.6.scaffold784.1 | ACC oxidase-4 like | 1.22 | 1.41 |
| CA.PGAv.1.6.scaffold793.14 | ACC oxidase-1 like | 1.18 | 1.52 |
| CA.PGAv.1.6.scaffold630.30 | ACC synthase-2 like | 1.09 | 1.48 |
| CA.PGAv.1.6.scaffold688.1 | glycine-rich protein 5-like | -1.76 | -1.28 |
| CA.PGAv.1.6.scaffold484.97 | ABC transporter B family member 11 | -2.66 | -3.15 |
