# Supplementary material for: Ethylene emitted by viral pathogen-infected pepper (Capsicum annuum L.) plants is a volatile chemical cue that attracts aphid vectors
Source: Front Plant Sci. 2022 Sep 29;13:994314. doi: 10.3389/fpls.2022.994314 (PMC9559363; doi:10.3389/fpls.2022.994314)
Supplement: Supplementary Figure 1 — Hierarchical tree graph of overrepresented GO terms for upregulated DEGs in response to CMV infection. [file Presentation_1.pptx]

## Slide 1
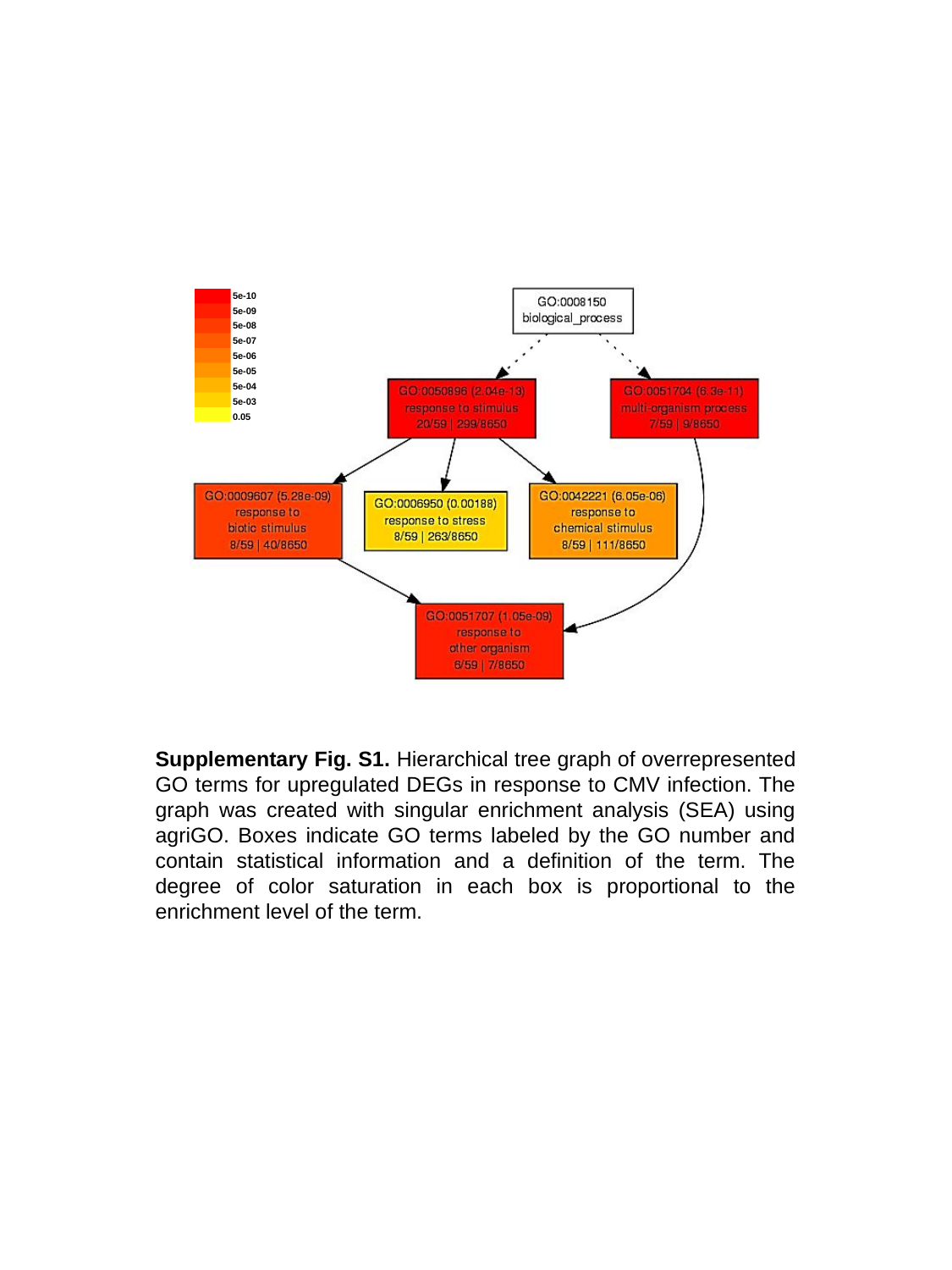

Supplementary Fig. S1. Hierarchical tree graph of overrepresented GO terms for upregulated DEGs in response to CMV infection. The graph was created with singular enrichment analysis (SEA) using agriGO. Boxes indicate GO terms labeled by the GO number and contain statistical information and a definition of the term. The degree of color saturation in each box is proportional to the enrichment level of the term.
